# Decreases in heart rate variability are associated with postoperative complications in hip fracture patients

**DOI:** 10.1371/journal.pone.0180423

**Published:** 2017-07-25

**Authors:** Gernot Ernst, Leiv Otto Watne, Frede Frihagen, Torgeir Bruun Wyller, Andreas Dominik, Morten Rostrup

**Affiliations:** 1 Department of Anaesthesiology, Kongsberg hospital, Kongsberg, Norway; 2 Section of Cardiovascular and Renal Research, Oslo University Hospital, Oslo, Norway; 3 Institute of Clinical Medicine, University of Oslo, Oslo, Norway; 4 Department of Behavioural Sciences in Medicine, Institute of Basic Medical Sciences, University of Oslo, Oslo, Norway; 5 Oslo Delirium Research Group, Department of Geriatric Medicine, Oslo University Hospital, Oslo, Norway; 6 THM University of Applied Sciences, KITE, Giessen, Germany; University of Minnesota, UNITED STATES

## Abstract

**Background:**

To explore relevant associations between deviations in linear and nonlinear heart rate variability (HRV) scores, and short-term morbidity and mortality in patients undergoing hip-surgery after a fracture.

**Methods:**

165 patients with hip fractures being admitted for surgery at two hospitals were included in a prospective cohort study. A short-term ECG was recorded within 24 hours of arrival. 15 patients had to be excluded due to insufficient quality of the ECG recordings. 150 patients were included in the final analysis. Linear parameters were calculated in time domain: standard deviation of NN intervals (SDNN), root mean square of successive differences (rMSSD); and frequency domain: Total Power (TP), High Frequency Power (HF), Low Frequency Power (LF), Very Low Frequency Power (VLF), and the ratio of LF/HF. Postoperative outcome was evaluated at the time of discharge. This included occurrence of pneumonia, overall infection rate, stroke, myocardial infarction, and all-cause mortality.

**Results:**

Patients experiencing complications had significantly lower rMSSD (p = 0.04), and TP (p = 0.03) preoperatively. Postoperative infections were predicted by decreased VLF preoperatively (p = 0.04). There was a significant association between pneumonia and LF/HF<1 (p = 0.03). The likelihood ratio to develop pneumonia when LF/HF < 1 was 6,1.

**Conclusion:**

HRV seems to reflect the general frailty of the patient with hip fracture and might be used to identify patients in need of increased surveillance or prophylactic treatment.

## Introduction

Due to demographic changes, an increasing number of hip fractures can be expected in the years to come [1] although age specific incidence is decreasing in some reports [2]. Surgery is necessary in these patients, but there is a relevant perioperative risk. A one-year mortality of about 25% has been observed [3–5]. It has been assumed that after one year, the mortality rate approaches that of age- and sex-matched controls [6], but in another study increased mortality was observed up to six years [7]. Heart failure [3,8], anaemia [4,9] chronic pulmonary disease [3,7], renal disease [3,8] and dementia [5,10] have been identified as risk factors for poor outcome. Over-all, at least 30 risk factors have been identified, mostly with moderate predictive value [11]. Better risk estimation is therefore needed. Heart rate variability (HRV) is an inexpensive and simple option.

HRV was introduced in a clinical context fifty years ago as beat-to-beat variation of the foetal ECG and was associated with distress before other detectable symptoms [12]. In 1987, Kleiger [13] demonstrated a possible role of HRV in predicting mortality after acute myocardial infarction. Since then, HRV has been investigated as a risk marker in cardiology, intensive care, traumatology, neurology, psychiatry and many other fields [14].

HRV has recently been proposed as a helpful, non-invasive, bedside, low-cost monitoring tool to evaluate autonomic dysfunction and its potential implications for preoperative assessment and early detection of complications like sepsis [15]. In the perioperative field, HRV has been used to predict preoperative instability [16], to monitor anaesthesia [17] and to estimate postoperative major cardiac events and mortality [18]. It is, however, far from being an established tool for anaesthetists.

Moreover, HRV has not only been associated with changes in the autonomic nerve system, it has increasingly also been considered as a parameter associated with general stability or fragility of the physiological system. Several studies have shown that HRV may change earlier than other parameters indicating increased instability e.g. inflammatory mediators detected in blood [19,20], alterations in endocrinological systems [21–23], and cognitive performance [24,25].

HRV might therefore reflect general physiological performance, and Goldberger et al. [26] suggested that increased regularity of signals may represent a decomplexification through illness, based on the notion that complex physiological systems with several parallel regulatory mechanisms increase stability, and that stability is associated with complex patterns in time series like heart beats [26]. Reduced HRV is a predictor of general mortality [27]. An association between decreased HRV and frailty in the elderly, defined as a state of critically impaired homeostasis that results in heightened vulnerability to stressors, has also been described [28].

Heart rate variability is obtained by measuring the RR-intervals in ECG recordings. Indices of the variability can be calculated by different algorithms in time domain and frequency domain [29]. A report from The Task force of the European Society of Cardiology recommends that one in *time domain* measures standard deviation of beat-to-beat intervals (SDNN), and root mean square of successive differences (rMSSD). In *frequency domain* one determines the frequency bands Total Power (TP), Very Low Frequency (VLF), Low Frequency (LF), High Frequency (HF), and the ratio of LF/HF [30].

Our study is based on the assumption that HRV might reflect general physiological performance, and that reduced HRV might predict postoperative complications in patients after hip fracture. While there have been numerous associations between HRV and cardiovascular events like myocardial infarction and stroke, it has also been suggested that decreased HRV is associated with the occurrence of infections and instability in general. Thus, in our study protocol, we decided to look at the most common complications associated with the postoperative course of hip fractures.

In the present prospective cohort study, relevant associations between deviations in linear and nonlinear HRV-scores, and short-term morbidity and mortality in patients admitted with an acute hip fracture were explored.

## Material and methods

90 patients with hip fractures admitted to Kongsberg hospital between 2008 and 2013 were invited to participate in the study. In addition, 75 patients admitted to Oslo University Hospital with a hip fracture between 2010 and January 2012 were also included. These patients were participating in a randomized controlled trial which evaluated the effect of orthogeriatric care on cognitive function four months after surgery in hip fracture patients [5]. Hemodynamically unstable patients, patients where it was impossible to obtain a 5 or 10-minute ECG signal (e.g. due to confusional state), patients having undergone surgery the last month, cancer patients, patients with hip fractures due to high energy trauma, and patients considered moribund at admission, were excluded. In Oslo, written informed consent was obtained from the patients or substitute decision-makers if patients did not have the capacity to consent. Only patients capable of giving written consent by themselves were recruited at Kongsberg.

A 5-10-minute ECG signal was recorded within 24 hours after arrival preoperatively and digitalized.

Heart rate was obtained by a Biocom 3000 ECG recorder (Kongsberg) and a Biocom 4000 ECG recorder (Oslo). The Biocom 3000 and 4000 ECG interface units use dry silver/ silver chloride ECG electrodes attached to two fingers of the right and the left hand, respectively. Participants were asked to relax for 5 minutes. Afterwards, they were connected to the ECG, and a continuous ECG signal was obtained over 10 minutes (Kongsberg) or 5 minutes (Oslo). Linear parameters (time domain: SDNN, rMSSD; frequency domain: HF, LF, VLF, LF/HF) were calculated by a Heart Rhythm Scanner—Version 2.0 –(Biocom Technologies–U.S.A). Both signal measurement and processing was done according to international recommendations [30].

We analyzed HRV both in time domain and frequency domain. Time domain analysis measures the intervals between successive normal cardiac cycles. SDNN (the standard deviation of the NN intervals) reflects all the cyclic components responsible for variability in the period of recording and correlates strongly with total power (TP) of the frequency domain. rMSSD (root mean square successive difference) is calculated by drawing the square root of the mean value of the squared NN intervals [31]. In healthy persons, the rMSSD value is 27 ± 12 ms [32]. It estimates high-frequency variations in heart rate and correlates accordingly mostly with HF in the frequency domain. Changes in this parameter might show a decreased parasympathetic tone and discordance in sympathovagal activity.

Frequency domain (power spectral density) analysis describes the periodic oscillations of the heart rate signal, decomposed at different frequencies and amplitudes, and provides information on the amount of their relative intensity (termed variance of power) in the sinus rhythm of the heart [33]. It is calculated with help of power spectral density by the fast Fourier transformation [30]. Frequently reported indices are TP (total power), VLF (very low frequency power, < 0.003–0.04 Hz), LF (low-frequency power, 0.04–0.15 Hz), HF (high-frequency power, 0.15–0.4 Hz), and the LF/HF ratio. It is recommended not to calculate VLF values from recordings lasting five minutes or less because VLF has a cycle period of 20 seconds to 5 minutes [30]. The measurement period should be at least twice as long as the cycle duration [34]. We therefore used only time series of 10 minutes (from the Kongsberg group) for calculation of VLF.

All ECGs were manually edited according to the Task force of the European Society of cardiology [30]. If containing more than 30% pathological QRS-complexes, the patients' data were excluded.

The sample size was estimated according to reference values reported in the work of Sztajzel et al. (2004)[33]. The calculation was based on mortality as the most important outcome. If we assume a mortality between 3 and 8% according to Dahl [35], 150 patients would be sufficient to test the hypothesis that there is a significant association between linear HRV-measurements and mortality. However, we also addressed other postoperative incidents than mortality, most of which occur more frequently. A total of 165 patients were finally included.

In the statistical analysis, we used the independent samples T test for univariate analysis and ANOVA for multivariate analysis. For nominal data, the Chi-Square test or Fisher's exact test were used, as appropriate. In case of very different group sizes (in the case of postoperative pneumonia) we used the nonparametric Mann-Whitney-U-test. Likelihood ratio was calculated by dividing sensitivity by 1 –specifity. Statistical analyses were run by the Statistical Package for Social Sciences (SPSS), release 18.0.3 (September 2010).Values are given in mean +/- SEM if not otherwise stated.

We tested in particular SDNN, rMSSD and different cut-off values of the parameters LF/HF (< 1.0, <1.5, <2.0) and SDNN (< 70, < 50) which have been reported in earlier studies [18,36–39].

Postoperative outcome was evaluated at the time of discharge by going through the medical records and the discharge letter. This included presence of postoperative infections (pneumonia and urinary tract infections), stroke, myocardial infarction, and all-cause mortality.

Pneumonia was diagnosed according to the presence of a new infiltrate on chest radiograph and the presence of one or several of the following acute respiratory signs or symptoms: cough, sputum production, dyspnea, core body temperature exceeding 38.0°C, auscultatory findings of abnormal breath sounds and rales, or leukocyte count greater than 10 x 10^9^ or less than 4 x 10^9^ cells L^–1^ (38). Urinary infection was diagnosed in case of clinical symptoms (fever, dysuria) and bacteriuria. Myocardial infarction was diagnosed by a specialist of internal medicine according to the following criteria: increase of patient's plasma of cardiac troponin (cTn) with at least one cTn measurement greater than the 99(th) percentile of the upper normal reference limit during symptoms of myocardial ischemia; new significant electrocardiogram (ECG) ST-segment/T-wave changes or left bundle branch block; the development of pathological ECG Q waves; or new loss of viable myocardium or regional wall motion abnormality identified by an imaging procedure [40]. Stroke was diagnosed according to the national guideline and international recommendations [41].

The study protocol was reviewed and approved of the Regional Committee for Medical and Health Research Ethics of Southern Norway (11.1.2008, S-07307b) and the Data Protection Officer of Oslo University Hospital.

## Results

165 patients were included in the current study, 90 from Kongsberg and 75 from Oslo. 41 (25%) had an established diagnosis of coronary heart disease, 62 (38%) arterial hypertension, and 31 (19%) COPD. Most frequently used medication included beta blockers, diuretics and statins. Patients taking betablockers had a heart rate of 75.7 (±1.7) compared to 79.8 (±1.0) at patients without beta blockers (p = 0.036). Detailed background data are outlined at Table 1, whereas results of blood tests are summarized in Table 2. 15 patients had to be excluded due to insufficient quality of the ECG recordings. 150 patients were included in the final analysis.

**Table 1:** Patient characteristics.

	All subjects		Kongsberg		Oslo	
	N	%	N	%	N	%
Number of patients	165	100	90	55	75	45
Mean age	80.9+/- 0.8		79.0 +/- 1.1		83.2 +/- 1.1	
Female	123	74.5	66	73.3	57	76
Coronary heart disease	41	25	23	26	18	24
Hypertension	62	38	33	37	29	39
Diabetes type I	7	4	3	3	4	5
Diabetes type II	15	9	10	11	5	7
COPD	31	19	17	19	14	19
Beta blockers	47	28	26	29	21	28
AT2-antagonists	23	14	13	14	10	13
ACE	27	16	21	23	6	8
Calcium antagonists	22	13	8	9	14	19
Digitalis	6	4	1	1	5	7
Nitrate	7	4	5	6	2	3
Diuretics	37	22	17	19	20	27
Cortisone	13	8	7	8	6	8
Statins	37	22	23	26	14	19
Sedatives	25	15	8	9	17	23
Antidepressives	35	35	15	17	20	27
Antiepileptics	7	4	4	4	3	4

**Table 2:** Initially blood sample results (Mean +/- SEM).

	All subjects	Kongsberg	Oslo
Hb	12.3 (±0.12)	12.3 (±0.16)	12.4 (±1.6)
Sodium	138 (±0.28)	138 (±0.38)	139 (±0.38)
Potassium	4.1 (±0.04)	4.0 (±0.05)	4.1 (±0.05)
Creatinine	82.6 (±2.65)	85 (±3.63)	80 (±3.88)
CRP	25 (±3.46)	22 (±3.82)	28 (±6.06)

One in three patients experienced at least one complication (Table 3). Seven percent developed pneumonia, and two percent died before discharge. Due to low numbers of myocardial infarctions and stroke, these patients were not analysed separately, but were included in the analysis of over-all complications.

**Table 3:** Postoperative complications.

	All patients		Kongsberg		Oslo	
	N	%	N	%	N	%
All complications	55	33.0	18	20.0	37	49.3
Death	4	2.4	1	1.1	3	4.0
Pneumonia	14	8.5	9	10	5	6.7
Urinary Tract Infection	16	9,8	4	4.4	12	16.0
Myocardial infarction	3	1.8	2	2.2	1	1.3
Stroke	2	1.2	1	1,1	1	1.3

**Fig 1:**
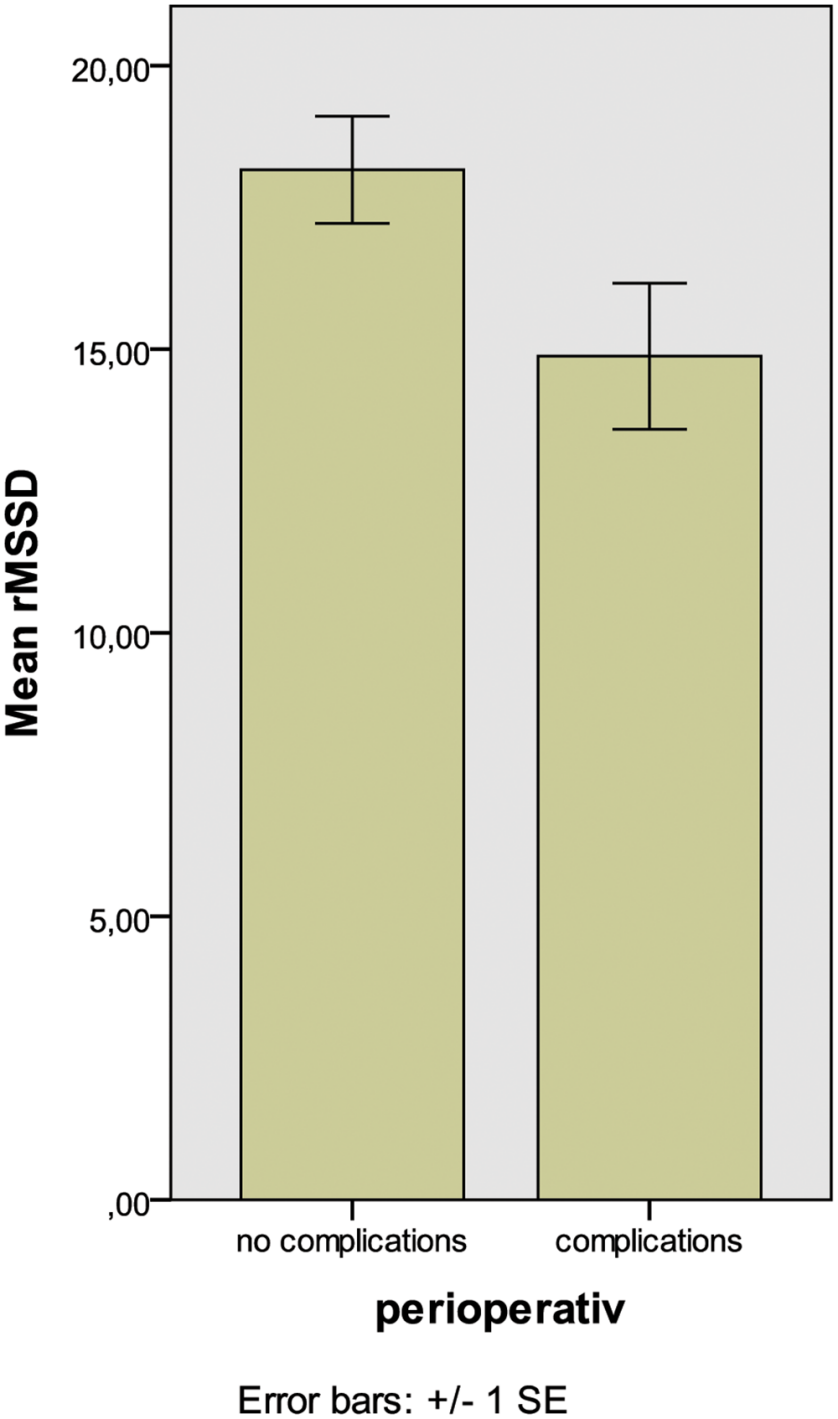
Association between preoperative rMSSD and postoperative complications (p = 0.043).

There were no differences in HRV parameters comparing patients with and without beta blockers. Patients with complications had significantly lower rMSSD and TP preoperatively (p = 0.043 and 0.03, respectively, Fig 1) compared to patients without. The likelihood ratio to develop complications in case of rMSSD < 10 was 4.9.

**Fig 2:**
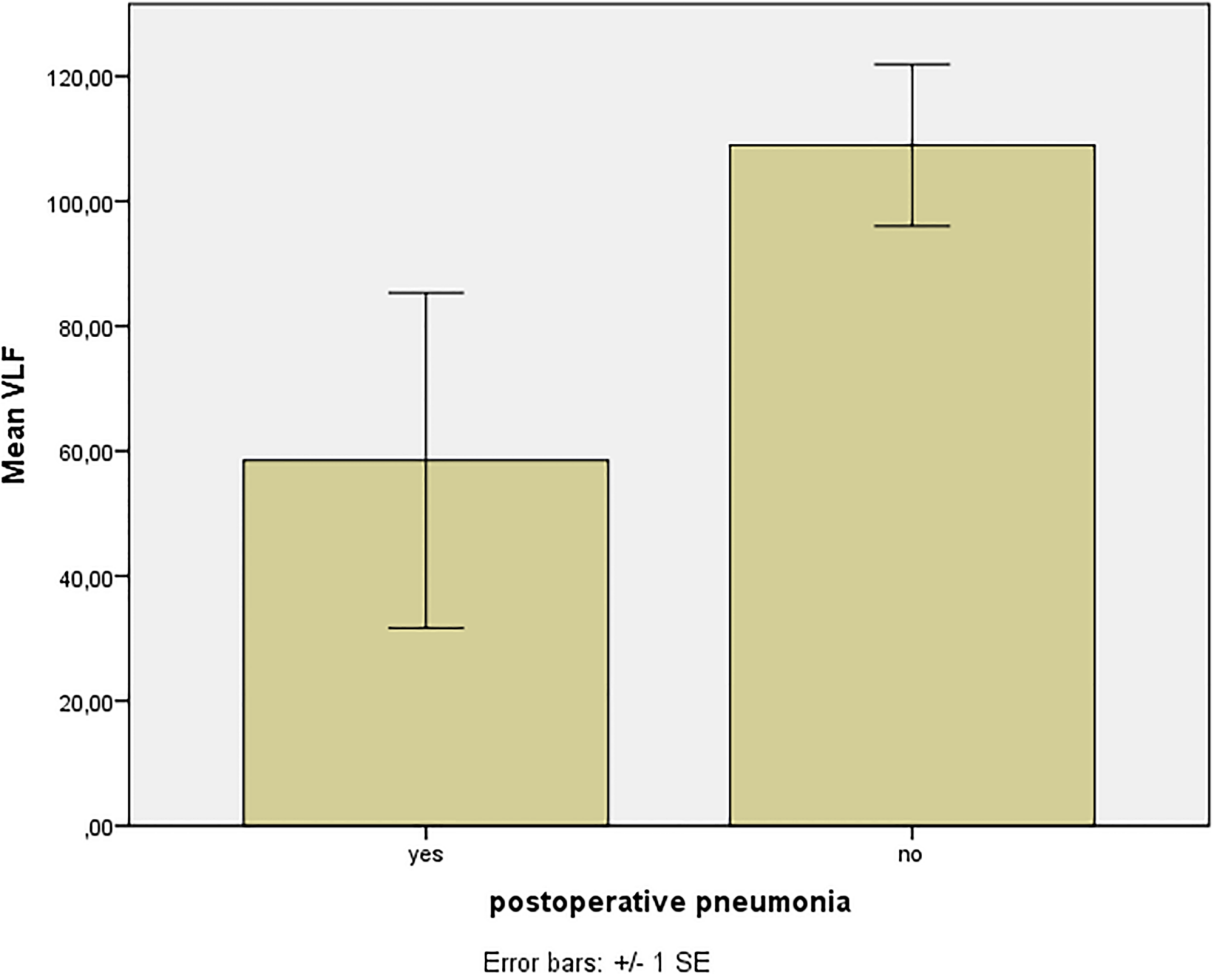
Association between preoperative VLF and postoperative pneumonia (p = 0.04).

**Fig 3:**
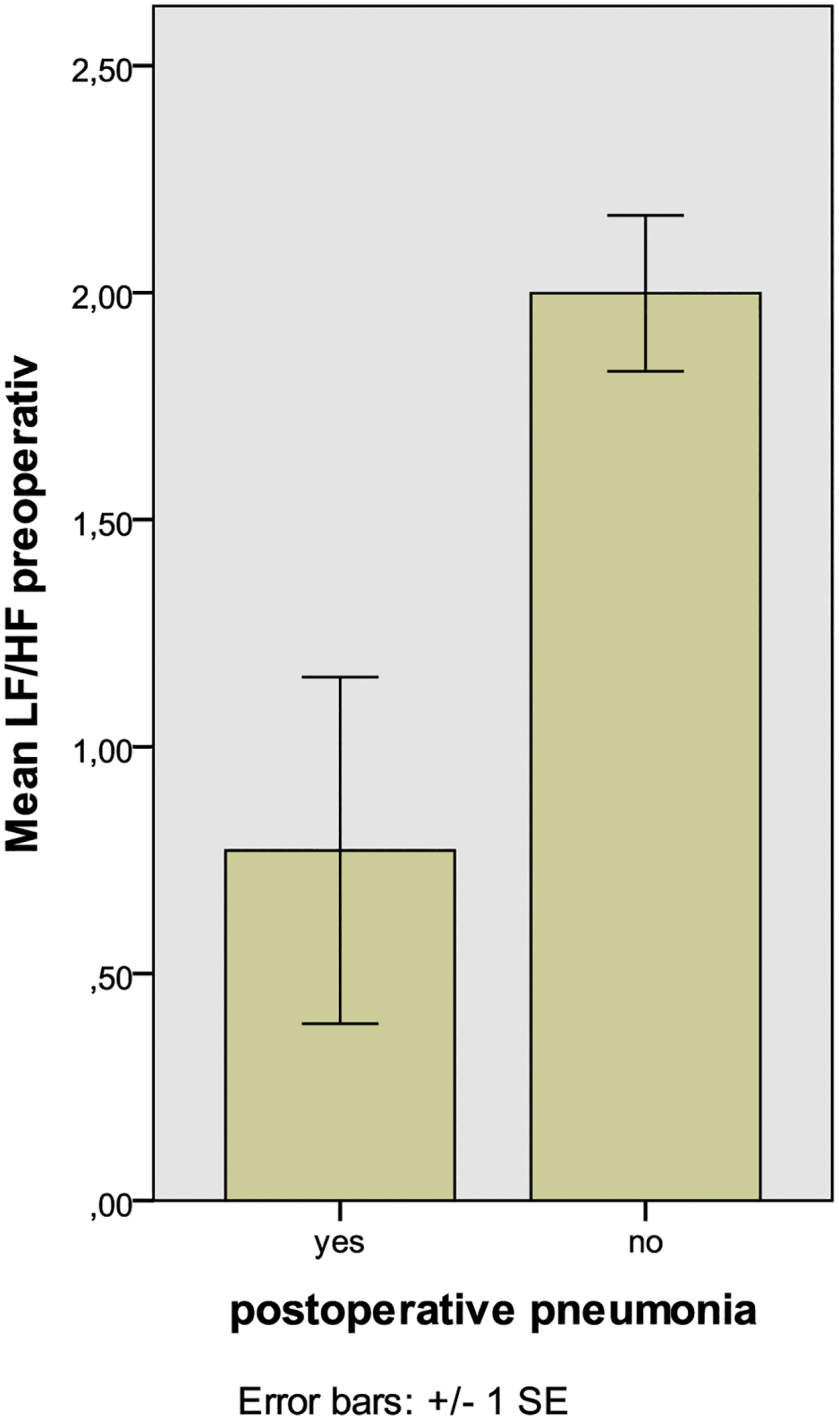
Association between LF/HF < 1 and postoperative pneumonia (p = 0.017).

Patients that experienced postoperative infections (pneumonia and urinary tract infection) had significantly lower VLF preoperatively, compared to patients without such complications (p = 0.04, Table 4 and Fig 2). In patients with postoperative urinary tract infections there was also decreased VLF (p = 0.02), and we found a tendency towards lowered VLF in patients with postoperative pneumonia (p = 0.091). There was a significant association between Pneumonia and LF/HF<1 (p = 0.031, Fig 3). The likelihood ratio to develop pneumonia in case of LF/HF < 1 was 6.1. In addition, we found a tendency to general complications for patients with LF/HF < 1 (p = 0.073). All results are summarized in Table 4.

**Table 4:** HRV parameters of patients with complications, infections in general, pneumonia and urinary tract infections. (Mean +/- SEM).

	No complications (n = 95)	Overall complications (n = 55)	Infections in general (n = 30)	Pneumonia (n = 14)	Urinary Tract Infections (n = 16)
SDNN	36.7 ± 2.8	37.0 ± 5.6	34.4 ± 6.6	43.6 ± 13.0	27.1 ± 4.5
rMSSD	18.2 ± 0.9	14.9 ± 1.3*	14.6 ± 1.6	16.2 ± 2.6	13.6 ± 2.0
TP	1082 ± 282	2525 ± 740*	1753 ± 779	2918 ± 1583	733 ± 393
HF	52.4 ± 7.9	54.1 ± 14.3	54.6 ± 17.0	62.8 ± 31.5	49.1 ± 19.8
LF	134.7 ± 23.1	196.5 ± 56.3	230.6 ± 82.3	245.0 ± 129.4	219.8 ± 110.0
VLF	110.0 ± 12.3	76.4 ± 32.6	44.8 ± 18.6*	58.5 ± 26.8	17.4 ± 7.7*
LF/HF	1.98 ± 0.19	1.70 ± 0.29	1.50 ± 0.37	0.77 ± 0.38*	2.17 ± 0.57

* = p < 0.05 compared to patients without complications

SDNN was not associated with general complications, pneumonia or postoperative death, neither in dichotomous data analysis with SDNN < 20, 50 or 70 or in the ANOVA model.

## Discussion

In this study of patients with hip fracture, we found significant associations between HRV-values and postoperative complications. Decreased TP and rMSSD, but not SDNN were associated with increased overall postoperative complications. Furthermore, there were decreased VLF and reduced LF/HF in patients with postoperative infections in general, and with postoperative pneumonia and urinary tract infections assessed separately. The current study is the first identifying a clear association between HRV parameters, especially low VLF and postoperative infections like pneumonia or urinary tract infections.

The subjects in our study showed the same characteristics as patients in other studies, with similar prevalence of cardiological and other diseases [42]. All patients were fasting at least six hours before the operation and sleep rhythm is frequently disturbed due to pain and immobility. It is possible that fasting or sleep deprivation might have influenced HRV. In addition some parameters differ depending on the time of the day [43]. Longer lasting dietary restriction can lead to an increase in HF and decrease in LF parameters [44]. Shorter restriction can lead to a moderate decrease of rMSSD [45]. Since all these confounders can be assumed similar in all patients, with or without postoperative complications, we doubt that they will systematically influence our results.

Patients taking beta blockers had a small, but significantly reduced heart rate compared to patients not taking beta blockers. There were, however, no differences in any HRV parameters. This is in contradiction to some earlier reports. The original guidelines mention a possible influence of beta-blockers, but do not recommend separate analysis of patients with and without beta blockers [46]. In several reports, beta blockers were associated with increased HRV parameter, like TP, HF, LF and VLF [47] or rMSSD and SDNN [48]. Most of these earlier studies have been conducted with patients with heart failure, but also studies including healthy participants have shown increased HRV parameters like SDNN, rMSSD, LF HF and TP[49]. Other studies, however, showed decreased LF and LF/HF and increased HF [50] or no effect [51] Experiments indicate that improvement of cardiac autonomic control measured by HRV after beta blockade could be explained by a change of heart rate only[52]. The lack of differences in HRV parameters between patients with and without beta-blockers in the current study, is probably related to the small difference in heart rate.

Patients included at Kongsberg hospital had a lower complication rate compared to patients included at Ullevål hospital. All patients were followed according to the study protocol including outcome parameters, but patients included at Ullevål hospital were also taking part in a study where a group of patients was allocated to special geriatric care (5). It is possible that patients at Ullevål hospital were more carefully monitored as a consequence of this study contributing to a higher rate of observed complications. The quality of monitoring could also have differed between the two sites because one of the sites was a university hospital, while the other was a much smaller provincial hospital.

Due to practical circumstances, we recorded ECG for 10 min in Kongsberg, and 5 min in Oslo. According to previous studies there seems to be no relevant difference in HRV assessments of five or ten minutes [53].

In the present study, we focussed on HRV as a possible preoperative tool to estimate the risk of postoperative complications. An association of pathological changes in the autonomic nervous system and worse outcome, e.g. in diabetic patients with advanced autonomous neuropathy, was established already decades ago [54]. Furthermore, a model that included myocardial perfusion scanning, D-Dimers *and* HRV (Holter monitoring over 24 hours) in 297 patients, showed a sensitivity and specificity of 84 and 80% respectively in predicting postoperative cardiac events in patients undergoing peripheral arterial surgery, including amputations [55]. Another model was developed in eighty patients with coronary artery disease with a planned bypass operation. Holter monitoring, plasma neuropeptides and catecholamines pre- and postoperatively were included. 36.3% of patients developed atrial fibrillation postoperatively, and they had a significant lower HF and LF/HF ratio. HF decreased in both groups postoperatively. Neither neuropeptides nor catecholamines differed between the groups [56].

A decreased rMSSD, as observed in our study in patients ahead of developing complications, may indicate a lower parasympathetic activity. Decreased rMSSD has previously also been associated with immunologic changes. Hs-CRP > 3 is associated with lower rMSSD compared with hs-CRP < 1 [57]. We observed also decreased Total Power (TP) in patients with complications. TP is usually associated with SDNN and reflects all cyclic components responsible for variability in the period of recording [30]. TP is for instance reduced in patients with diabetic autonomic neuropathy [58]. Reduced TP has also been observed in an early phase of developing hypertension [59].

The VLF-component appears to indicate a more healthy function, and an increase in resting VLF power may reflect increased sympathetic activity, though its origin is controversial [31]. It is not used as often as other parameters, but has been more strongly associated with clinical outcome than LF in some studies [60]. Decreased VLF is often associated with increased inflammatory parameters like CRP, Il-6 and WBC [61] and might therefore be associated with perturbations in the immune system. Proposed mechanisms responsible for altered VLF are as diverse as thermoregulatory processes, the Renin-Angiotensin system [62], hemodynamic feedback delays, mechanical and central neural effects of breathing patterns, a central oscillator, spinal reflexes, and vascular autorhythmicity [63]. Newer experimental work suggests that VLF is influenced by the stimulation of afferent sensory neurons in the heart, which in turn activate various levels of the feedback and feed-forward loops in the heart and involve extrinsic cardiac ganglia. VLF might be an intrinsic rhythm that does not only signal health and well-being, but contributes to the stability of this system [31]. High CRP is associated with low HRV. Reduced SDNN and VLF were the best predictors of high CRP [64,65]. A recent review concluded that there is clear evidence of an association between on-going subclinical inflammation and reduced heart rate variability [20].

LF/HF has been investigated as a tool to early detect sepsis [19,37]. Korach et al. observed increased development of sepsis when LF/HF was low, with a likelihood ratio of 6.47 with LF/HF < 1 [37]. No patient developed sepsis in our study, but we found a clear association between LF/HF < 1 and postoperative pneumonia, with a likelihood ratio of 6.1, which may support this earlier finding. In addition we found a tendency to over-all complications in patients with LF/HF < 1 (p = 0.073). HRV changes in neonates and adults appear sometimes up to 24 hours before other infection symptoms [19,66,67]. Parameters like VLF and LF/HF ratio might therefore be an interesting screening tool in this patient group.

It is interesting to note that SDNN was not predicting any complication. This is in contradiction to reports where SDNN has been associated with increased morbidity and mortality in various clinical populations [36,38,39]. Assuming an increased inflammatory response, we would expect SDNN to decrease [64,65]. Most clinical studies analysed SDNN with a long follow-up and it’s prognostic value might be limited in our patient group. The prognostic value of SDNN in patients with hip fractures has not been studied before.

Risk prediction models for specific postoperative complications like delirium [68] and pneumonia [69] have been published in hip fracture patients. To our knowledge, no model exists for a general estimation of postoperative complications in this patient group. If our results can be confirmed, heart rate variability could be an interesting contribution to the assessment of postoperative risks in this elderly population.

## Conclusion

Our study showed an association between lower rMSSD, TP and general complications, an association between low VLF and postoperative infections and between LF/HF<1 and postoperative pneumonia. HRV might be a feasible surrogate factor reflecting the general frailty of the patient and might be used to identify patient groups that need increased surveillance and prophylactic treatments.

## Supporting information

S1 File (SAV)
